# A MOBILE MUSIC APPLICATION FOR CAREGIVERS OF PERSONS WITH DEMENTIA: A USABILITY TESTING STUDY

**DOI:** 10.1093/geroni/igac059.1770

**Published:** 2022-12-20

**Authors:** Cassidy Doyle, Debra Dobbs, Kelly Wiechart, Rohan Wadhwa, Hongdao Meng

**Affiliations:** University of South Florida, Tampa, Florida, United States; University of South Florida, Tampa, Florida, United States; KellyW Consulting, Tampa, Florida, United States; University of South Florida, Tampa, Florida, United States; University of South Florida, Tampa, Florida, United States

## Abstract

More than 11 million Americans spend an average of 26.3 hours caring for a person with dementia (PWD). Managing behavioral and psychological symptoms of dementia (BPSD) is a significant source of caregiver stress. Music-based interventions (MBIs) have been shown to be a pleasant activity enjoyed by both PWD and their caregivers, with the potential to help alleviate BPSD. However, current commercial music streaming services are not designed for families coping with dementia due to subscription and/or network connection requirements. This mixed-methods usability study tested caregivers’ experience with an open-source Android application (MUsic to Support Engagement and Resilience, or MUSER) as a potential platform for home-based MBI delivered by caregivers of PWD. In-lab and in-home testing sessions were conducted with 6 caregivers (2 former and 4 current caregivers, 1 male and 5 females). Semi-structured interviews were conducted before and after the in-home testing period between July and November 2021 to better understand User Experiences (UX). Family caregivers had a mean age of 57.8 (SD=26.2, range 20-77). The main themes identified from the interviews included ease of use, device and music preferences, and prior experience with technology. Overall, users reported high satisfaction and would recommend the application to other caregivers. Users reported that listening to music on the app elicited positive emotional effects (e.g., calmness and relaxation). The MUSER mobile music application can be used by caregivers to deliver MBI in the community. Future research should test whether a caregiver-delivered MBI would be efficacious in reducing BPSD among PWD.

